# Primary cardiac lymphoma: two cases and a review of literature

**DOI:** 10.1186/s13019-015-0348-0

**Published:** 2015-10-30

**Authors:** Karolis Jonavicius, Kestutis Salcius, Raimundas Meskauskas, Nomeda Valeviciene, Virgilijus Tarutis, Vytautas Sirvydis

**Affiliations:** 1 Faculty of Medicine, Vilnius University, Vilnius, Lithuania; 2 Department of Cardiovascular Medicine, Faculty of Medicine, Vilnius University, Vilnius, Lithuania; 3 National Centre of Pathology, Affiliate of Vilnius University Hospital Santariskiu Clinics, Vilnius, Lithuania; 4 Department of Radiology, Nuclear Medicine and Physics of Medicine, Faculty of Medicine, Vilnius University, Vilnius, Lithuania; 5 Vilnius University Faculty of Medicine Centre of Cardiac Surgery, Santariskiu g. 2, Vilnius, 08661 Lithuania

**Keywords:** Primary cardiac lymphoma, Heart tumour, Large B cell lymphoma

## Abstract

**Background:**

Primary cardiac lymphoma is one of the rarest tumours of the heart. It belongs to the extra-nodal non-Hodgkin’s lymphomas. The most common type of this tumour is diffuse large B cell lymphoma. Usually, right atrium and right ventricle are involved. This tumour is fatal unless diagnosed and treated in time. In this article two female patients who were diagnosed with primary cardiac lymphoma and treated at our clinic are described. The first patient went to remission after the treatment, while the second patient died. The goals of this article are to show the difficulties of diagnosing and treating this disease, the role of cardiac surgery in its treatment and to raise awareness of this disease.

**Case reports:**

In this article two female patients who were diagnosed with primary cardiac lymphoma and treated at our clinic are described. The first patient went to remission after the treatment, while the second patient died.

**Conclusions:**

Primary cardiac lymphoma is a very rare disease. At the moment the most effective treatment is chemotherapy. Palliative surgery may be necessary to correct hemodynamics when venous blood flow to the lungs is disturbed.

## Background

Primary cardiac lymphoma (PCL) is a rare malignant disease. A. Johri et al. state that PCL composes just 1.3 % of all cardiac tumours [1]. L. Zhong et al. write that PCL composes only 0.5 % of all extranodal lymphomas [2]. The most common histological type of PCL is large B cell lymphoma [2, 3]. PCL is fatal, unless it is diagnosed and treated in time [4]. Patients usually die within few months after being diagnosed with PCL [5–7]. In this article two patients diagnosed with PCL and treated at our clinic are presented.

## Case reports

**Fig. 1:**
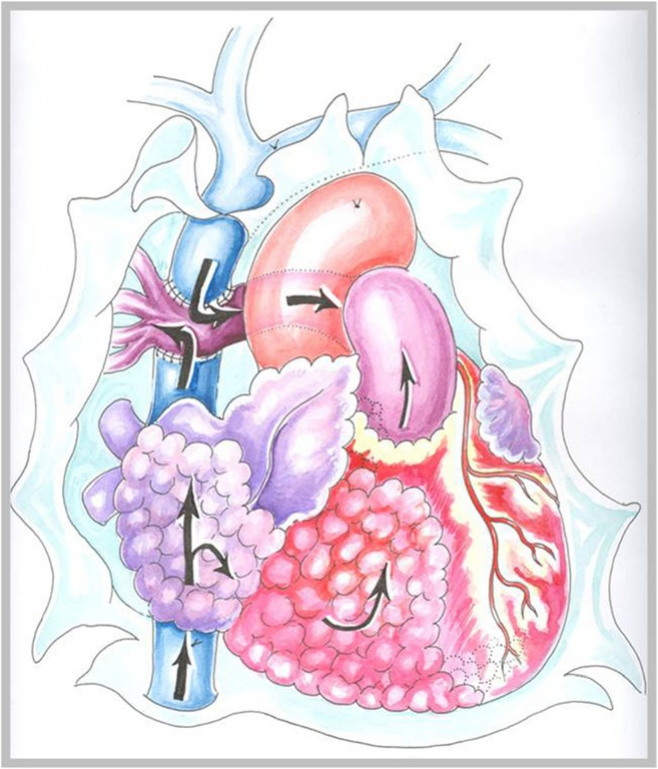
Schema of the procedure performed for the first patient (drawing by O. Barysaite). Black arrows indicate venous blood flow through the right side of the heart

Patient 1, a 48 year old female, who had a history of progressive heart failure, dyspnoea at rest and cyanosis for three weeks prior to hospitalization. The patient had no complaints of fever above 38 C, night sweating or loss of more than 10 % of body weight over a period of six or less (B symptoms). On the day of hospitalization a transthoracic echocardiography (TTE) was performed. An inhomogeneous tumour was found in both atria. It was almost closing the orifice of the tricuspid valve (the diameter of the tricuspid valve orifice was 2.38 cm and the diameter of the part of the tumour closing the orifice was 2.25 cm) and penetrating the left ventricle via the interventricular septum. Complete blood count (CBC) and coagulation profile test (CPT) were within normal limits. The patient was immunocompetent and human immunodeficiency virus (HIV) tests were negative. Due to a rapid and severe heart failure an open chest operation was performed, during which a biopsy of the tumour was taken. Fast intra-operative analysis of the specimen revealed that the tumour was malignant. Because the tumour was inoperable and the patient’s hemodynamics was unstable, a decision was made to perform a Fontan procedure as a palliative method in order to stabilize the patient’s condition and to provide time for chemotherapy. The superior vena cava (SVC) was anastomosed to the right pulmonary artery (RPA) (Fig. 1). This way venous blood flow was redirected to the lungs and obstructed right heart was bypassed. After the surgery, the patient’s condition improved. She was extubated on the second day after the surgery. Breathing was spontaneous and arterial blood oxygen saturation (SaO_2_) was 92 %. A detailed histological exam of the tumour revealed that it was diffuse large B cell lymphoma. There were no evidence of extra-cardiac lymphoma present, thus the final diagnosis was primary cardiac lymphoma. When the patient recovered from the surgery, she decided to continue her chemotherapy treatment at a foreign oncology clinic. To the best of our knowledge, the patient went into remission after chemotherapy.

**Fig. 2:**
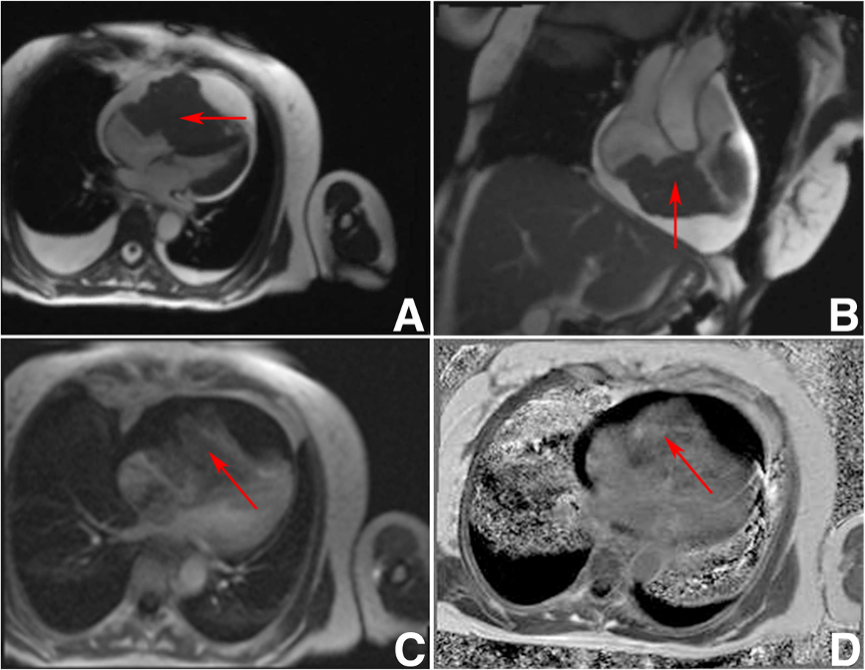
Preoperative patient 2 Heart MRI. **a** 4 chambers GRE view. Hypointensive masses visible in right ventricle, right atrium and right atrial appendage (red arrow). The tumour is obstructing the right ventricle and compressing the interventricular septum, and the left ventricle. **b** 2 chambers GRE view. Hypointensive masses in the right ventricle and right atrium (*red arrow*). **c** 4 chambers view perfusion sequence. Perfusion is seen in the masses (*red arrow*). **d** 4 chambers view. Non homogenous gadolinium enhancement is visible (*red arrow*). All images were taken during diastole

**Fig. 3:**
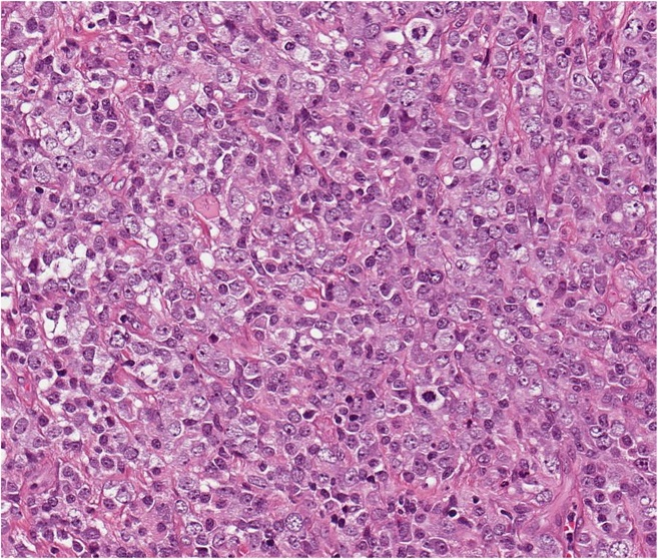
Patient 2 tumour histology. Diffuse myocardial infiltration with large B cells (Hematoxylin-eosin stain, original magnification × 200)

Patient 2, a 64 year old female was diagnosed, with acute nonspecific idiopathic pericarditis, and was referred to our clinic. She had a history of heart failure for six months prior to hospitalisation. On the day of arrival she presented with a decompensated heart failure. Tachypnoea, hepatomegaly and ascites were present. The patient had no complaints regarding the B symptoms. Arterial blood pressure was 100/70 mmHg. CBC and CPT were within normal limits. HIV tests were negative, the patient was immunocompetent. TTE revealed a severe dilatation of the right atrium and right ventricle, a large tumour in the right side of the heart and fluid accumulation in the pericardial sac. In order to specify the diagnosis a pericardiocentesis, cardiac magnetic resonance imaging (MRI) and percutaneous transvenous biopsy (PTVB) of the tumour were performed. The pericardial fluid exam showed that it was transudate with no cells. The MRI revealed a tumour in the right atrium appendage, which was invading the right atrium and ventricle and visceral pericardium. The large tumour (10 x 7.2 x 8 cm) was obstructing the right ventricle, the IVS was pushed towards the left ventricle and slightly interfering with its function (end systolic volume of the LV was 26 ml, end diastolic volume of the LV was 53 ml and LV EF was 52 %, cardiac index was 1. 8 l/min/m^2^) (Fig. 2). PTVB of the tumour showed mild myocardial fibrosis. 11 days later the patient’s condition started to worsen. An open chest biopsy of the tumour was performed. A fast intra-operative histological exam of the tumour revealed that it was malignant. Because the tumour was inoperable, a decision to perform a Fontan procedure was made. It was intended to stabilise the patient’s hemodynamics. After the surgery the patient’s general condition was satisfactory. She was extubated twelve hours later. Breathing was spontaneous and SaO_2_ was 90 %. Eight hours later, the patient’s condition began to worsen. She was re-intubated. Asystole developed and cardio-pulmonary resuscitation was initiated. All means to save her life were ineffective. The patient passed away. Detailed histological exam of the tumour revealed that it was a diffuse large B cell lymphoma (immunophenotype: non-germinal centre, CD20-positive, CD10-negative, BCL2-positive, BCL6-positive, MUM1-positive, CD23-negative, CD30-negative, EBER-negative) (Fig. 3).

## Discussion

There are no unified criteria when this tumour can be called primary cardiac lymphoma. L. Zaharia and P. Gill state that PCL can be diagnosed when the tumour involves the pericardial space and myocardium [8]. On the other hand, Curtsinger et al. state that PCL can only be diagnosed when there is cardiac lymphoma without any other evidence of lymphoma on autopsy [9]. Both of our patients were diagnosed with PCL’s, because histological exams of both tumours showed they were diffuse large B cell lymphomas. And there were no evidence of a tumour present in any other organ of both patients.

PCL is a rare disease. It composes only 1.3 % of all cardiac tumours and 0.5 % of all extranodal lymphomas [1, 2]. The largest review of case-reports and case-series was performed by Petrich et al. It included 197 cases of PCL reports from 1947 – 2009. The authors reported that this disease is more common in the elderly age. The median age of the patients is 63 years), although it ranged from 9 to 90 years (but only 5 patients were younger than 17 years old) [10]. The reported male to female ratio is 2(3):1 [2, 10]. The symptoms of PCL are nonspecific. It can manifest as a heart rhythm disturbance, episodic syncope, vena cava superior syndrome, respiratory distress [2] or even as a restrictive cardiomyopathy [1, 2, 11]. However, the most common symptoms are dyspnoea, constitutional complaints (fever, chills, sweats and weight loss), chest pain, heart failure and pericardial effusion [2, 10]. PCL is more likely to involve the right heart (both atrium and ventricle alike), but there are cases were only the left heart was involved [1, 2, 10, 12–15].

Patients who are suspected of having a PCL must be examined thoroughly. The examination must include echocardiography, heart computed tomography or MRI study, but the final diagnosis can be made only after a histological evaluation of the tumour is obtained. Usually PTVB with transesophageal echocardiographic (TEE) control is used to obtain the specimen [14, 15]. D. Jurkovich et al. has successfully used a combined fluoroscopic imaging and TEE for PTVB [16]. However, in our second case PTVB failed to provide any clinically significant data.

PCL treatment is combined of surgery and chemotherapy. The literature indicates, that when treating with chemotherapy alone 61 % of patients have a remission, while surgery alone has no effect to the outcome [15]. However, surgery is the only method which can provide time for chemotherapy to have a therapeutic effect, especially when hemodynamics is disturbed. If the tumour is localized in the right ventricular outflow tract (RVOT) and partially or fully occludes the outflow tract, the prognosis is worse [3]. Even in the case of RVOT obstruction surgical treatment does not change the outcome prognosis. As stated by Y. H. Jung et al. it is necessary to diagnose PCL as early as possible, to use intensive treatment with new chemotherapy drugs in order to achieve better outcomes for the patients [3]. K. W. Chen et al. and later A. Habertheuer et al. reported successful recovery of patients after surgical removal of the tumour and postoperative chemotherapy [4, 17].

It was impossible to remove the tumour of either patient. They underwent a surgery because of the obstructed blood flow to the lungs. The main goal of the procedure was to provide patients with time for chemotherapy to have a therapeutic effect. Our first patient survived the procedure and went to remission after chemotherapy. Our second patient, died on the first day after surgery. It is unknown why the outcome of both patients was different. It is possible that the difference in outcomes is related to the elderly age and far more developed initial heart failure of the second patient.

## Conclusions

Primary cardiac lymphoma is a very rare disease. At the moment the most effective treatment is chemotherapy. Palliative surgery may be necessary to correct hemodynamics when venous blood flow to the lungs is disturbed.

## Consent

Written informed consent for publication could not be obtained from patient 1 and patient’s 2 next of kin despite all reasonable attempts. Every effort has been made to protect the identity of our patients and ensure their anonymity.

## Abbreviations

B symptoms: unexplained fever above 38 °C, night sweating, loss of more than 10 % weight loss over a period of six or less months
CBC: complete blood count
CPT: coagulation profile test
HIV: human immunodeficiency virus
MRI: magnetic resonance imaging
PCL: primary cardiac lymphoma
PTVB: percutaneous transvenous biopsy
RPA: right pulmonary artery
RVOT: right ventricle outflow tract
SaO2: arterial blood oxygen saturation
SVC: superior vena cava
TEE: transesophageal echocardiography
TTE: transthoracic echocardiography
